# Mitochondrial genome sequence of black paradise flycatcher (Aves: Monarchidae) and its phylogenetic position

**DOI:** 10.1080/23802359.2016.1181996

**Published:** 2016-07-08

**Authors:** Soo Hyung Eo, Junghwa An

**Affiliations:** a Department of Forest Resources, Kongju National University, Chungnam, Republic of Korea;; b Animal Resources Division, National Institute of Biological Resources, Incheon, Republic of Korea

**Keywords:** Avian mtDNA, mitogenome, phylogeny, *Terpsiphone atrocaudata*

## Abstract

We generated the complete mitochondrial genome of the black paradise flycatcher (*Terpsiphone atrocaudata*; Family: Monarchidae), an ecologically important insectivorous bird in Asian forest ecosystems. The mitogenome was 16,984 bp in length and consisted of 13 protein-coding genes, 22 tRNAs, two rRNAs and a control region. Gene composition and arrangement in the mitogenome were similar to those of related families Corvidae and Laniidae available in GenBank. However, *tRNA^Ala^* was located between *COXII* and *ATP8* genes in the mitogenome of *T. atrocaudata* while *tRNA^Lys^*, was in the same location in the mitogenomes of Corvidae and Laniidae. The phylogenetic tree based on the mitogenomes of *T. atrocaudata* and the related families supported that Monarchidae was the sister taxa to the clade of Laniidae and Corvidae. The mitogenome of *T. atrocaudata* will be a valuable genetic resource for phylogenetic analyses and implication of conservation and management of the species.

Black paradise flycatcher (*Terpsiphone atrocaudata* Eyton, 1839) also called Japanese paradise flycatcher is a passerine bird breeding mainly in the Korean Peninsula, Japan and Taiwan and migrating to China and Southeast Asia (Mizuta 1998; Del Hoyo et al. 2006; Kim et al. 2010, 2011). The species is an ecologically important insectivore in deciduous or mixed forest ecosystems of the breeding distribution but, unfortunately, the bird is suspected to be in rapid decline by habitat loss and, therefore, listed as Near Threatened of IUCN Red List (Del Hoyo et al. 2006; BirdLife International 2015).

Approximately one hundred species including *T. atrocaudata* belong to the monarch flycatcher family, Monarchidae (Gill & Donsker 2015). The systematic position of the family Monarchidae has been largely dependent on results of DNA–DNA hybridization (Sibley & Ahlquist 1990) and data from multilocus nuclear genes (Barker et al. 2002, 2004). However, comprehensive surveys of mitochondrial genome (mitogenome) for the phylogenetic placement of the family are lacking. Complete mitogenome plays a significant role in discussion of phylogenetic relationships (Eo and An. in press; Eo & DeWoody 2010) and implication of conservation and management (Eo et al. 2010) but, to our knowledge, no complete mitogenome has been reported from the family Monarchidae. Here, we sequenced the complete mitogenome of *T. atrocaudata* in order to provide further molecular resources for the biology, phylogenetic analysis and conservation of the species.

The specimen of *T. atrocaudata* was collected by CGRB (Conservation Genome Resource Bank for Korean Wildlife; CGRB3837), South Korea. We extracted genomic DNA from muscle tissue of the bird and prepared genomic libraries with an insert size of 300–700 bp. A HiSeq2500 sequencing platform (Illumina, San Diego, CA) generated about 5.8 million sequence reads corresponding to about 0.81 Gbp from genomic DNA of the black paradise flycatcher. We assembled the sequence reads into contigs using the default parameters in Newbler ver3.4 (Illumina, San Diego, CA).

The complete mitogenome sequence of a *T. atrocaudata* was 16,984 bp in length (GenBank accession no. KT901458) with 44.3% of the GC content. The mitogenome was composed of 13 protein-coding genes, 12S and 16S ribosomal RNAs, 22 transfer RNAs and a non-coding control region. The start codon was ATG in all protein-coding genes with an exception of the COXI (GTG). Gene composition, length and arrangement in the mitogenome were similar to those of related families Corvidae and Laniidae available in GenBank. However, *tRNA^Ala^* was located between COXII and ATP8 genes in the mitogenome of *T. atrocaudata* while *tRNA^Lys^*, was in the same location in the available mitogenomes of the Corvidae and Laniidae.

A neighbor-joining (NJ) tree was reconstructed using 13 protein-coding genes of the *T. atrocaudata* mitogenome and all available complete mitogenomes of crows and allies in GenBank, with those of a *Vireo olivaceus* mitogenome as an outgroup (Figure 1). Our NJ tree revealed that the family Monarchidae (*Terpsiphone*) was the sister taxa to the clade of the Laniidae (*Lanius*) and the Corvidae (*Corvus*, *Nucifraga*, *Garrulus*, *Pica*, *Podoces*, *Urocissa*, *Cyanopica* and *Pyrrhocorax*). The result is similar to those of the phylogenetic trees based on multilocus nuclear genes (Barker et al. 2002, 2004). The complete mitogenome of *T. atrocaudata* will be a valuable genetic resource for phylogenetic relationships and implication of conservation and management of the species.

**Figure 1.**
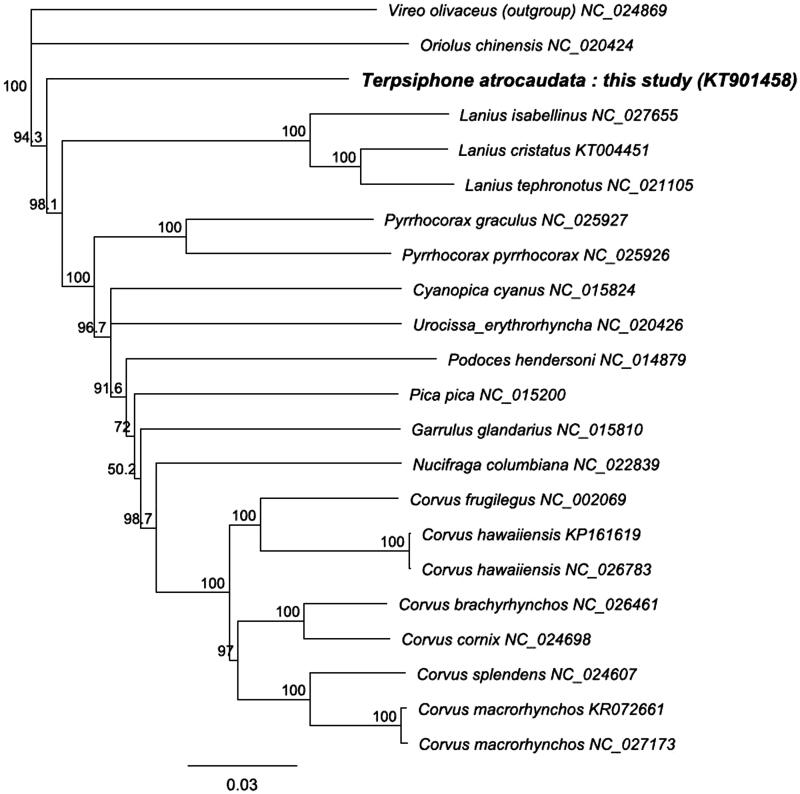
Neighbor-joining tree based on 13 protein-coding genes from 20 crows and allies, *Terpsiphone atrocaudata* (KT901458) we sequenced and *Vireo olivaceus* as an outgroup. Numbers on branches represent bootstrap supports (1000 replicates).
